# Effects of different strains fermentation on nutritional functional components and flavor compounds of sweet potato slurry

**DOI:** 10.3389/fnut.2023.1241580

**Published:** 2023-08-24

**Authors:** Long Pan, Cun-Jin Zhang, Zhe Bai, Ying-Ying Liu, Yu Zhang, Wei-Zhi Tian, Yu Zhou, Yuan-Yuan Zhou, Ai-Mei Liao, Yin-Chen Hou, Guang-Hai Yu, Ming Hui, Ji-Hong Huang

**Affiliations:** ^1^Henan Provincial Key Laboratory of Biological Processing and Nutritional Function of Wheat, School of Biological Engineering, Henan University of Technology, Zhengzhou, China; ^2^College of Food and Biological Engineering, Henan University of Animal Husbandry and Economy, Zhengzhou, China; ^3^School of Food and Pharmacy, Xuchang University, Xuchang, China; ^4^State Key Laboratory of Crop Stress Adaptation and Improvement, College of Agriculture, Henan University, Kaifeng, China; ^5^Food Laboratory of Zhongyuan, Henan University of Technology, Zhengzhou, China

**Keywords:** sweet potato slurry, fermentation, nutritional components, gas chromatography-mass (GC-MS), flavor substances

## Abstract

In this paper, we study the effect of microbial fermentation on the nutrient composition and flavor of sweet potato slurry, different strains of *Aspergillus niger, Saccharomyces cerevisiae, Lactobacillus plantarum, Bacillus coagulans, Bacillus subtilis, Lactobacillus acidophilus*, and *Bifidobacterium brevis* were employed to ferment sweet potato slurry. After 48 h of fermentation with different strains (10% inoculation amount), we compared the effects of several strains on the nutritional and functional constituents (protein, soluble dietary fiber, organic acid, soluble sugar, total polyphenol, free amino acid, and sensory characteristics). The results demonstrated that the total sugar level of sweet potato slurry fell significantly after fermentation by various strains, indicating that these strains can utilize the nutritious components of sweet potato slurry for fermentation. The slurry’s total protein and phenol concentrations increased significantly, and many strains demonstrated excellent fermentation performance. The pH of the slurry dropped from 6.78 to 3.28 to 5.95 after fermentation. The fermentation broth contained 17 free amino acids, and the change in free amino acid content is closely correlated with the flavor of the sweet potato fermentation slurry. The gas chromatography-mass spectrometry results reveal that microbial fermentation can effectively increase the kinds and concentration of flavor components in sweet potato slurry, enhancing its flavor and flavor profile. The results demonstrated that *Aspergillus niger* fermentation of sweet potato slurry might greatly enhance protein and total phenolic content, which is crucial in enhancing nutrition. However, *Bacillus coagulans* fermentation can enhance the concentration of free amino acids in sweet potato slurry by 64.83%, with a significant rise in fresh and sweet amino acids. After fermentation by *Bacillus coagulans*, the concentration of lactic acid and volatile flavor substances also achieved its highest level, which can considerably enhance its flavor. The above results showed that *Aspergillus niger* and *Bacillus coagulans* could be the ideal strains for sweet potato slurry fermentation.

## Introduction

Sweet potato is an extremely popular food item. This tuber plant has a pleasant flavor, and many individuals enjoy it. The production of sweet potatoes in China has surpassed 85% of the globe. The production of sweet potatoes have reached 51 million tonnes in 2022 (1). The cultivation area and output of sweet potato are only lower than those of rice, wheat, and corn among China’s food crops, ranking fourth (2). It is adaptable, resistant to drought, produces a large yield, has a wide range of applications, and is both edible and feed. Sweet potatoes can also be processed into starch and utilized as raw materials in the light industrial sector. It is crucial to boost grain output and improve people’s living standards to raise the production of sweet potatoes according to local requirements (3).

Furthermore, to a small amount of fresh food, certain sweet potatoes are currently utilized for biological fermentation to make fuel ethanol. In contrast, others are used for primary processing, such as sweet potato vermicelli, sweet potato starch, dried sweet potato, sweet potato juice, etc. (4, 5). During primary processing, a significant quantity of nutrients cannot be utilized and are wasted. According to the reports, sweet potatoes are rich in dietary fiber, protein, amino acids, polysaccharides, and phenolic compounds, etc., and their nutritional worth is acknowledged worldwide (6, 7). If sweet potatoes can be treated aggressively to acquire additional nutrients, their use will be substantially enhanced. Microbial fermentation has historically been one of the most prevalent food processing methods (8–10). Under the influence of beneficial microorganisms, carbohydrates, lipids, and proteins in food materials are decomposed and transformed into distinctive flavors and other nutrients. Domestic and international researchers use lactic acid bacteria, bacillus, and edible fungi, etc. to ferment fruit and vegetable juice to increase its nutritional and sensory qualities (11–13). However, the utilization of these microbes in the fermentation of sweet potato slurry has not been reported.

Therefore, this study employs sweet potato slurry as its basic material and ferments it with various microorganisms. To provide data support and a theoretical framework for the preparation of beverages from industrial fermentation slurry, the microorganisms suitable for the fermentation of sweet potato slurry were chosen through a thorough comparison of the functional nutritional components (protein, total dietary fiber, sugar components, organic acid, total phenols, and free amino acids) and sensory characteristics (GC-MS) of the fermented slurry.

## Materials and methods

### Preparation of sweet potato slurry

Fresh, pest- and disease-free sweet potatoes were peeled (Longshu No.9, purchased from Yonghui Supermarket). Then, they were combined with water in a 1:2 (w/w) ratio in the pulping wall-breaker (SP301S, Zhejiang Supor Co., Ltd., Hangzhou, China). The slurry of sweet potato was kept at 95°C for 2 h after 10 U/g of high-temperature amylase (20,000 U/mL, provided by Shanghai Yuanye Bio-Technology Co., Ltd.) addition and then kept at 40°C for 3 h after 20 U/g amyloglucosidase (100,000 U/mL, provided by Shanghai Yuanye Bio-Technology Co., Ltd.). The sweet potato slurry was then split into 250 mL shake flasks containing 100 mL of liquid. The liquid is then pasteurized and stored at 4°C for future fermentation studies.

### Microorganism

This study employed these strains, *Aspergillus niger* (*A.n*), *Saccharomyces cerevisiae* (*S.c*), *Lactobacillus plantarum* (*L.p*), *Bacillus coagulans* (*B.c*), *Bacillus subtilis* (*B.s*), *Lactobacillus acidophilus* (*L.a*), and *Bifidobacterium brevis* (*B.b*). All these strains were preserved in our laboratory at −80°C.

### Seed culture of different microorganisms

YPD culture medium was purchased from Shanghai Yuanye Bio-Technology Co., Ltd. (Shanghai, China) and is majorly used for *S. cerevisiae* seed culture; MRS culture medium, purchased from Solarbio Biotechnology Co., Ltd. (Beijing, China) and it is mostly employed for the seed culture of other microorganisms. All media were autoclaved for 20 min at 115°C.

The seed culture medium was inoculated with different strains after sterilization, and then incubated at 37°C and 150 rpm/min for 24 h in the rotary shaker (HYL-C, Qiangle Experimental Co., Ltd., Taicang, China). Typically, 10% of the seeds were then transferred to a 250 mL shake flask containing 100 mL of sweet potato slurry and incubated for 48 h at 37°C and 150 rpm on a rotating shaker. Also, 10% sterile water was utilized as the inoculant in the control experiment. All fermentations were repeated three times.

### Measurement of total sugars and soluble sugars

The 3,5-dinitrosalicylic acid (DNS) colorimetric method was used to determine the total sugar concentration (8). High-Performance Liquid Chromatography (HPLC 1515, Waters, United States) was used to examine soluble carbohydrates (fructose, glucose, and trehalose) (14). Briefly, 1 mL of each sample was transferred to an injection vial and then loaded onto the autosampler of a chromatograph equipped with a sulfonated polystyrene divinylbenzene column (Aminex HPX-87C 300 mm × 7.8 mm, 9 μm; Bio-Rad Chemical Division, California) and a refractive index detector. The mobile phase consisted of acetonitrile: water (70:30) at a 0.6 mL/min flow rate. The injection volume was 20 μL. The column and detector were kept at 50°C.

### Measurement of pH and organic acids

The pH was measured using a pH electrode (FE28-Micro, Mettler Toledo, Zurich, Switzerland). According to Li et al., organic acids (acetic acid, citric acid, lactic acid, malic acid, and succinic acid) were analyzed using HPLC (15). Briefly, 1 mL of each sample was injected into the injection vial and placed on the autosampler of the chromatograph with a C18 column (150 × 4.6 mm, 5 μm; Agilent) and an ultraviolet detector. The mobile phase was 40 mM Na_2_SO_4_ (adjusted to pH 2.68 using CH_3_SO_3_H) at a 0.8 mL/min flow rate and injection volume of 10 μL. The column temperature was maintained at 25°C, and the UV detector’s wavelength was 210 nm.

The fermentation samples were centrifuged, and the supernatant was filtered through a 0.22 μm aqueous membrane. Identification and quantification were performed based on standard solutions retention times and calibration curves.

### Measurement of the content of protein and soluble dietary fiber

Using a BCA protein assay kit (Tiangen, Beijing, China) with bovine serum albumin as a reference, the protein concentration of fermentation broth was measured. The absorbance in reaction units was determined using the Synergy H4 Hybrid Microplate Reader (Biotek, Winooski, VT, United States) at room temperature.

Soluble dietary fiber was performed following the AOAC 991.43 method (16). The 50 mL of fermentation broth was centrifuged to extract the supernatant, then 5 mL of protease was added, and the mixture was agitated for 60 min. The reactant was centrifuged at 12,000 × g for 15 min. The supernatant was filtered using a 0.45 μM millipore filter (Jinteng Laboratory Equipment Co., Ltd., Tianjin, China) and precipitated by injecting four volumes of ethanol at 4°C for 24 h. The precipitate was again filtered, dissolved in distilled water, and freeze-dried as SDF.

### Measurement of total polyphenols

Total polyphenol content was determined using the technique described (10). One mL of each juice and Folin–Ciocalteu were mixed and reacted for 1 min, then 3 mL of 20% Na_2_CO_3_ was added to the mixture. The mixture was then reacted for 30 min at 50°C, and the absorbance was measured at 765 nm. The total polyphenol content was gallic acid equivalent (mg GAE/L).

### Measurement of free amino acid concentration

The FAA composition and content of various fermentation samples were analyzed using a fully automated amino acid analyzer L8900 (Hitachi, Japan) following the method of Liao et al. (17). The fermentation broth was centrifuged to extract the supernatant, then 400 μL supernatant was taken, 100 μL of sulfonic acid was added, and the mixture was uniformly mixed and stored at 4°C. After 1 hour, the supernatant was centrifuged at 12000 rpm for 5 min and diluted with PBS to an acceptable onboard concentration (estimated according to the protein content of the sample). The filtrate was collected through a 0.22 μm microporous aqueous membrane and evaluated with an automated amino acid analyzer.

### Determination of volatile compounds

The volatile compounds in fermentation samples were analyzed by a Headspace Solid-Phase Microextraction coupled with Gas Chromatography-Mass Spectrometer (HS-SPME-GC/MS) method described by Yang et al. (18) with certain modifications.

### Gas chromatography-mass spectrometry analysis

The GC-MS parameters were set as follows: The injector temperature was set at 300°C under the splitless mode. The initial oven temperature was 100°C and held for 1 min, then increased at a rate of 15°C/min to 300°C and held there for 5 min. The total running time was 16 min. Helium (99.999%) was the carrier gas with a 1.0 mL/min flow rate and a 6.0 mL/min blow-down flow rate. The EI source and mass transfer line temperatures were set to 230°C and 280°C, respectively. Selective ion monitoring (SIM) mode was selected with a solvent delay of 4 min. The mass spectrum was scanned by electron ionization (EI) mode at 70 eV to obtain a range of 35–500 m/z. The peak area ratio calculated the substance content (percentage).

### Statistical analysis

All studies were carried out in triplicate, and the data are shown as the mean ± standard deviation (mean ± S.D.). *T*-test used for significance analysis with GraphPad Prism 6.0 to confirmed the validity of the comparison tests; *p* < 0.05 was considered significant.

## Results and discussion

### Nutrient composition of sweet potato slurry fermented by different bacterial strains

Rich in nutrients, the sweet potato slurry can serve as a nitrogen-rich source for microbial development. Simultaneously, the microorganism-produced extracellular protease can hydrolyze the protein into small molecular oligopeptides and amino acids, supplying the product with abundant flavoring compounds (19). Compared with the unfermented sweet potato slurry, the soluble protein content was increased to a certain extent after fermentation (except for *S. cerevisiae*), among which the protein content increased the most significantly after fermentation by *B. coagulans* (Table 1), reached 52.05%. According to the results, *B. coagulans* produced a substantial amount of extracellular protein during the fermentation process, enhancing sweet potato slurry’s protein content. This was consistent with prior findings (1).

**Table 1:** Nutrient composition of sweet potato starch slurry fermented by different microorganisms.

Sample	Total protein (g/L)	SDF (mg/L)
N	8.28 ± 0.16^c^	42.42 ± 0.32^a^
*L.p*	10.42 ± 0.11^ab^	18.99 ± 0.23^c^
*L.a*	10.83 ± 0.21^ab^	15.41 ± 0.18^d^
*B.b*	9.29 ± 0.09^bc^	4.77 ± 0.65^e^
*A.n*	11.00 ± 0.14^a^	19.74 ± 0.22^c^
*B.l*	12.59 ± 0.12^a^	27.81 ± 0.37^b^
*B.s*	8.83 ± 0.11^c^	6.13 ± 0.15^e^
*S.c*	6.53 ± 0.07^d^	4.77 ± 1.25^e^

N: means inoculating an equal amount of sterile water as a control experiment; Different letters indicate significant differences at *p* < 0.05.

Dietary fiber is a material that cannot be digested and absorbed by the small intestine of humans (20). Fermented sweet potato slurry contained considerably less soluble dietary fiber (SDF) (Table 1). According to studies, dietary fiber can be fermented and metabolized by microorganisms to generate acids (21, 22).

### Changes of total phenol in sweet potato slurry after fermentation

**Figure 1:**
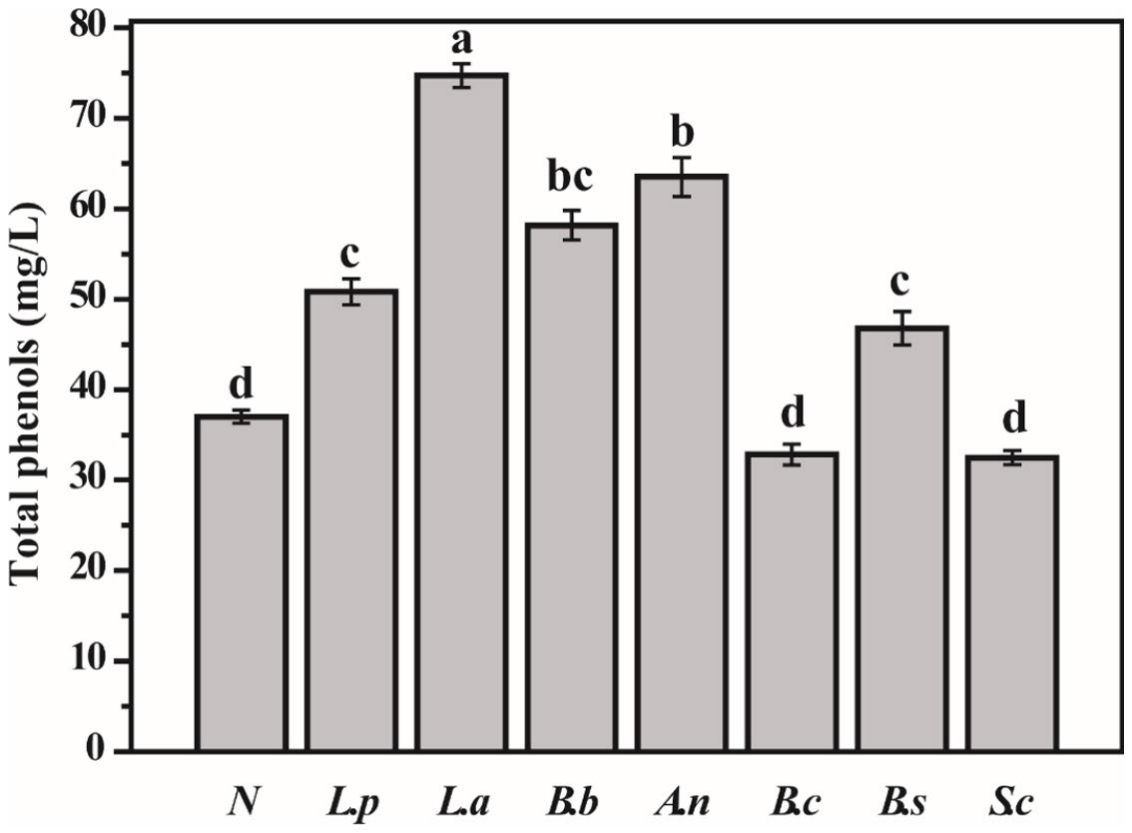
Determination of total phenols in fermented sweet potato slurry (Different letters indicate significant differences at *p* < 0.05. ND, not detected).

Phenols are bioactive compounds capable of ameliorating hypertension, hyperlipidemia, hyperglycemia, and inflammatory response (23). After fermentation, the total phenol content of sweet potato slurry increased considerably relative to unfermented sweet potato slurry (except for *S. cerevisiae* and *B. coagulans*). The total phenol concentration of the sweet potato slurry fermented with *L. acidophilus* and *A. niger* was 74.74 mg/L and 63.53 mg/L, respectively, while the unfermented sweet potato slurry had 37.02 mg/L (Figure 1). According to numerous studies, the microbial fermentation of fruit and vegetable juice can significantly enhance the overall phenol content of the fermentation broth (24–26). This may be because microorganisms synthesize carboxylase, tannase, and glycosidase during fermentation to facilitate polyphenols’ degradation and metabolic transformations (27). The data above indicate that *S. cerevisiae* does not affect the total phenol level of sweet potato slurry fermentations.

### Changes of total sugars and soluble sugars in sweet potato slurry after fermentation

The starch in the sweet potato slurry is degraded into glucose, fructose, and maltose through early enzymatic hydrolysis, thereby supplying energy for the growth and metabolism of microorganisms during the fermentation process. The metabolism and biotransformation of carbohydrates by microorganisms during fermentation considerably decreased the glucose level of fermented sweet potato slurry compared to unfermented sweet potato slurry (*p* < 0.05) (Table 2). This result is consistent with the changes in sugars during fermentation of most fruits and vegetables (28).

**Table 2:** Determination of total sugars in sweet potato slurry.

Sample	Total sugars (g/L)	Glucose (g/L)	Fructose (mg/L)	Sucrose (mg/L)	Maltose (mg/L)
N	56.32 ± 2.17^a^	48.74 ± 2.34^a^	297.7 ± 5.32^b^	597.32 ± 4.98^a^	ND
*L.p*	22.44 ± 2.25^cd^	20.18 ± 1.19^d^	380.62 ± 7.23^a^	287.77 ± 3.54^c^	ND
*L.a*	32.02 ± 2.77^b^	25.38 ± 1.52^c^	285.51 ± 5.18^b^	398.89 ± 4.93^b^	110.87 ± 3.23^a^
*B.b*	24.99 ± 1.15^c^	18.76 ± 2.05^d^	19.03 ± 1.05^e^	ND	ND
*A.n*	19.87 ± 1.31^d^	16.11 ± 1.17^e^	403.16 ± 4.24^a^	293.41 ± 5.34^c^	88.97 ± 2.77^b^
*B.c*	34.54 ± 2.78^b^	33.68 ± 1.22^b^	26.27 ± 1.57^d^	ND	ND
*B.s*	25.73 ± 1.29^c^	23.33 ± 1.34^cd^	88.25 ± 2.17^c^	ND	ND
*S.c*	3.06 ± 0.57^e^	1.52 ± 0.23^f^	85.27 ± 1.98^c^	132.88 ± 3.42^d^	ND

N: means inoculating an equal amount of sterile water as a control experiment; Different letters indicate significant differences at *p* < 0.05. ND, not detected.

### Changes of pH and organic acids in sweet potato slurry after fermentation

After fermentation by several bacterial strains, the pH value of sweet potato slurry was reduced to a certain level, with the greatest fall occurring in *B. coagulans* fermentation slurry (6.78 to 3.28) (Table 3). This is mostly because the amylase and lipase produced by the metabolism of *B. coagulans* during fermentation can convert sugars, lipids, and other compounds into organic acids and fatty acids, resulting in a considerable fall in pH (29). Simultaneously, after fermentation by several bacterial strains, the concentrations of lactic acid, acetic acid, citric acid, and succinic acid in the slurry increased significantly (*p* < 0.05). However, malic acid concentrations exhibited a decreasing trend, which was in consistent with Liang et al. (9), it means that malic acid can be used by these strains to produce other metabolites. The highest content of lactic acid was fermented by *B. coagulans* (2325.47 mg/L). The acetic acid was undetectable in the unfermented sweet potato slurry but reached 400.05 mg/L following *B. brevis* fermentation. *A. niger* produced the greatest concentration of citric acid (418.22 mg/L), indicating that *A. niger* has an abundant enzyme system that can convert glucose to make citric acid (30). The succinic acid was only detected after the fermentation of *A. niger*, *B. coagulans,* and *S. cerevisiae*. These organic acids can impart distinctive fermentation flavors to foods that have been fermented. The distinct sour flavor of lactic acid can improve food flavor, maintain the stability and safety of the product’s microorganisms, and make the taste gentle. Acetic acid can be absorbed by the blood, enter the liver for metabolism, and produce lipids and cholesterol; Citric acid functions as a flavoring agent and possesses antioxidant characteristics that increase the shelf life of food (31). Succinic acid, when absorbed by the human body, has a protective impact on organs. It can strengthen the body and enhance immune function (32, 33).

**Table 3:** Determination of pH and organic acids in sweet potato slurry.

Sample	pH	Lactic acid (mg/L)	Acetic acid (mg/L)	Citric acid (mg/L)	Malic acid (mg/L)	Succinic acid (mg/L)
N	6.78 ± 0.06^a^	67.1 ± 1.14^f^	ND	29.7 ± 0.32^f^	498.31 ± 2.67^a^	ND
*L.p*	5.64 ± 0.15^b^	403.22 ± 3.52^d^	100.63 ± 1.42^c^	150.62 ± 1.23^c^	396.14 ± 1.94^b^	ND
*L.a*	5.85 ± 0.12^b^	625.86 ± 2.82^c^	77.71 ± 1.09^d^	178.51 ± 2.18^b^	212.11 ± 1.55^d^	ND
*B.b*	5.81 ± 0.11^b^	84.021 ± 1.31^f^	400.05 ± 2.05^a^	8.03 ± 0.05^g^	364.09 ± 1.98^bc^	ND
*A.n*	5.95 ± 0.09^b^	886.62 ± 3.15^b^	23.11 ± 0.17^f^	418.22 ± 3.22^a^	101.22 ± 0.34^e^	205.72 ± 1.89^a^
*B.c*	3.28 ± 0.12^d^	2325.47 ± 5.77^a^	58.37 ± 1.22^e^	49.57 ± 0.67^e^	96.87 ± 0.74^e^	41.88 ± 0.74^b^
*B.s*	5.74 ± 0.13^b^	896.55 ± 2.09^b^	238.33 ± 2.34^b^	81.43 ± 0.65^d^	335.52 ± 1.52^c^	ND
*S.c*	4.37 ± 0.07^c^	220.17 ± 1.89^e^	60.52 ± 0.96^e^	90.26 ± 1.05^d^	325.63 ± 1.78^c^	189.25 ± 1.99^a^

N: means inoculating an equal amount of sterile water as a control experiment; Different letters indicate significant differences at *p* < 0.05. ND, not detected.

### Types and contents of free amino acids in sweet potato slurry after fermentation

Amino acids are one of the active macromolecules used in building biological organisms that can supply energy for life processes. Some microbes can create proteases and degrade macromolecular proteins into peptides and amino acids during fermentation. Therefore, the quantities of total amino acids and various amino acids in sweet potato slurry have altered significantly after fermentation. Compared with the unfermented sweet potato slurry, the total FAAs reached to 1819.74 mg/L with the fermentation of *B. coagulans*, which was 64.83% increased (Table 4).

**Table 4:** Content of FAAs in sweet potato slurry with the fermentation of different microorganisms (mg/L).

Amino acid	*N*	*L.p*	*L.a*	*B.b*	*A.n*	*B.c*	*B.s*	*S.c*
Asp	370.29 ± 2.17	32.82 ± 1.34	9.02 ± 0.33	9.71 ± 0.32	11.71 ± 0.02	469.04 ± 2.78	14.11 ± 0.12	11.09 ± 0.14
Thr	123.33 ± 2.32	18.43 ± 0.28	2.11 ± 0.02	13.33 ± 1.09	4.65 ± 0.03	77.57 ± 1.32	6.56 ± 0.07	8.44 ± 0.23
Ser	84.74 ± 1.52	33.87 ± 0.66	6.02 ± 0.15	9.64 ± 0.22	9.11 ± 0.02	102.23 ± 1.61	11.41 ± 0.08	15.71 ± 0.15
Glu	116.09 ± 1.44	61.43 ± 1.22	31.91 ± 1.11	26.85 ± 1.11	99.14 ± 1.32	168.86 ± 1.44	26.61 ± 0.22	22.76 ± 0.44
Gly	8.54 ± 0.11	8.71 ± 0.52	1.61 ± 0.02	3.32 ± 0.12	3.53 ± 0.04	172.25 ± 1.12	3.81 ± 0.02	5.31 ± 0.09
Ala	23.41 ± 0.31	48.37 ± 1.32	13.92 ± 032	34.21 ± 1.37	13.72 ± 0.12	115.34 ± 0.54	35.09 ± 0.44	4.95 ± 0.11
Cys	39.53 ± 1.08	57.77 ± 1.91	79.34 ± 1.67	22.72 ± 0.68	35.12 ± 0.32	34.04 ± 0.32	40.61 ± 0.92	35.65 ± 0.28
Val	58.13 ± 2.56	260.64 ± 1.42	187.82 ± 3.15	218.03 ± 3.22	173.33 ± 1.06	81.75 ± 0.25	160.34 ± 1.21	39.79 ± 0.33
Met	23.62 ± 0.41	59.04 ± 1.38	42.53 ± 1.08	34.85 ± 1.77	69.63 ± 0.32	23.56 ± 0.12	27.51 ± 0.19	17.11 ± 0.18
Ile	17.10 ± 0.22	19.36 ± 0.32	14.57 ± 0.22	18.23 ± 1.02	52.33 ± 0.88	26.81 ± 0.42	13.72 ± 0.22	2.23 ± 0.02
Leu	28.26 ± 1.02	73.18 ± 2.77	21.21 ± 1.31	41.83 ± 2.55	39.43 ± 0.12	57.75 ± 0.27	48.75 ± 0.44	6.51 ± 0.12
Tyr	58.51 ± 1.89	128.23 ± 2.35	183.11 ± 2.83	109.62 ± 0.88	45.25 ± 0.32	87.53 ± 0.44	103.32 ± 1.17	4.41 ± 0.08
Phe	105.24 ± 1.32	275.51 ± 1.17	295.81 ± 2.12	248.41 ± 1.54	154.14 ± 2.21	134.14 ± 1.81	233.72 ± 2.82	18.77 ± 0.44
Lys	20.04 ± 1.55	63.01 ± 0.32	62.56 ± 1.09	31.71 ± 0.32	23.13 ± 0.61	40.63 ± 0.38	41.81 ± 0.32	4.12 ± 0.22
His	19.97 ± 0.82	29.82 ± 1.02	19.31 ± 0.16	16.03 ± 0.12	2.21 ± 0.05	28.71 ± 0.08	18.05 ± 0.52	2.51 ± 0.08
Arg	6.93 ± 0.12	ND	ND	ND	ND	129.75 ± 1.18	ND	ND
Pro	ND	98.65 ± 0.51	162.54 ± 1.01	114.51 ± 1.22	8.07 ± 0.04	70.45 ± 0.17	96.67 ± 0.38	ND
T-FAA	1104.02 ± 3.23	1268.31 ± 3.51	1133.02 ± 2.77	952.61 ± 3.83	743.92 ± 3.12	1819.74 ± 4.15	881.64 ± 1.55	198.84 ± 1.84
UAA	506.92 ± 1.44	157.22 ± 1.77	103.44 ± 1.26	68.24 ± 0.54	133.97 ± 1.35	678.47 ± 2.17	82.53 ± 0.28	37.85 ± 0.19
SAA	344.91 ± 1.22	384.74 ± 1.87	319.45 ± 1.67	308.81 ± 1.21	184.92 ± 1.25	601.33 ± 2.58	290.41 ± 1.22	53.04 ± 0.94
BAA	259.64 ± 1.32	584.95 ± 2.56	576.43 ± 1.31	468.87 ± 2.18	362.78 ± 1.81	488.05 ± 2.51	444.95 ± 2.12	51.43 ± 0.88

Furthermore, being healthy nutrients, amino acids play a crucial function in flavor. Microorganisms can convert amino acids into fragrances peculiar to fruits and vegetables, such as fruit scent and fat aroma, through fermentation (34). According to different flavor approaches, free amino acids are classified as umami amino acids (UAA: including Glu, Asp., and Lys), sweet amino acids (SAA: including Thr, Ser, Gly, Ala, and Phe), and bitter amino acids (BAA: containing Tyr, Ile, Leu, Phe, Arg, His, and Met) (35). After fermentation by *B. coagulans*, the concentrations of UAA and SAA in unfermented sweet potato slurry increased to 678.47 mg/L and 601.33 mg/L, respectively. BAA level rises during fermentation (except in the case of *S. cerevisiae*), which may be related to the production of taste compounds during microbial metabolism (36). The modifications of these flavor amino acids can serve as a benchmark for the future regulation of flavor compounds in the food processing industry.

### Analysis of volatile components in sweet potato slurry fermented by different strains

**Figure 2:**
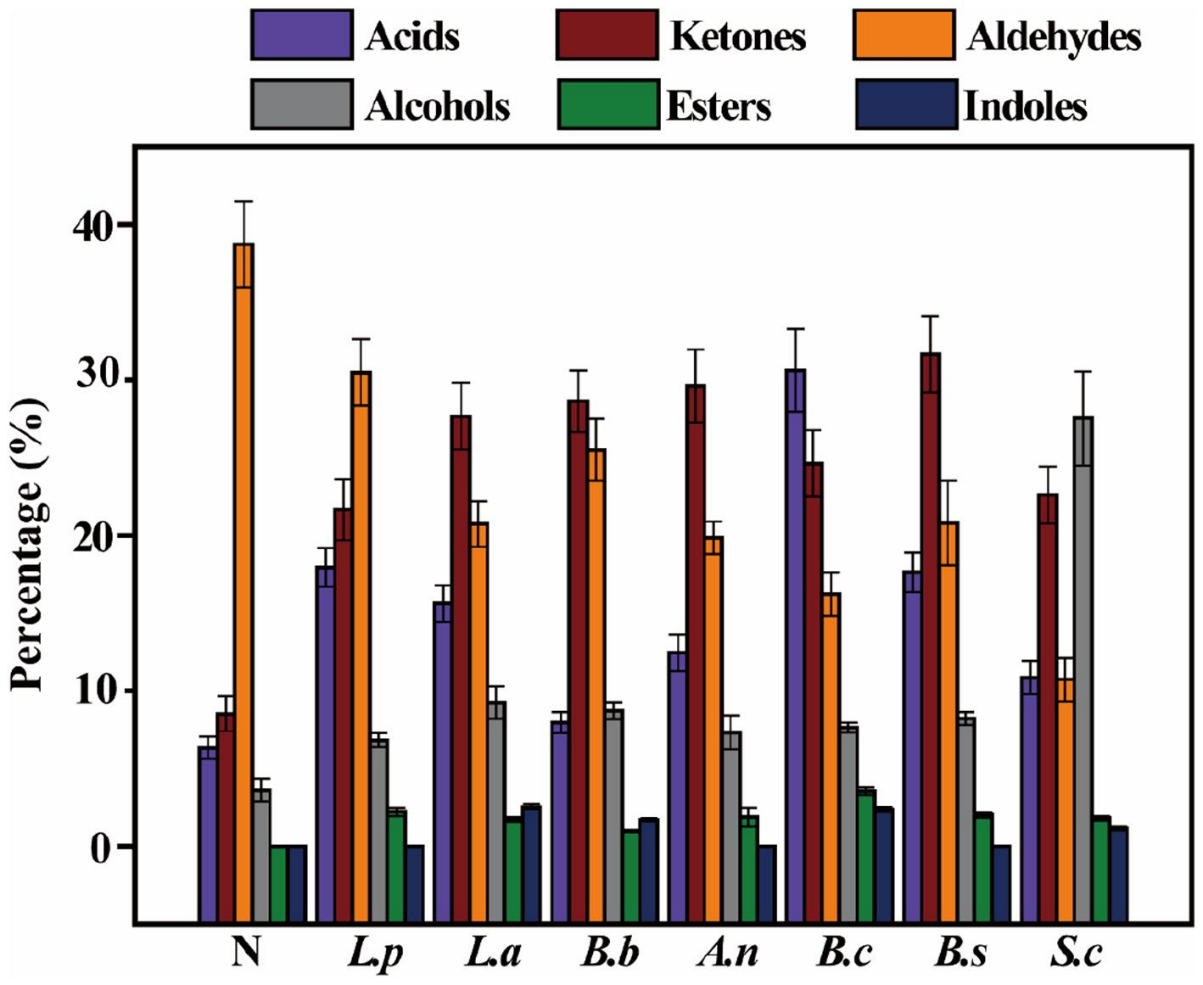
Changes of volatile compounds in sweet potato starch slurry fermented by seven different strains.

Flavor substances are the primary sensory component of all foods, imparting the most intuitive feeling to consumers. Using HS-SPME-GC-MS, 49 volatile chemicals were identified in various fermentation samples. There are 17 alcohols, 12 aldehydes, 5 acids, 6 esters, 6 ketones, and 3 indoles. As depicted in Figure 2, the components of volatile taste compounds in sweet potato slurry have experienced substantial modifications during bacterial fermentation. The percentage of flavor substances was the highest after fermentation by *B. coagulans*, which reached 86.55%.

The concentration of aldehydes was highest in unfermented slurry, reaching 38.72%, although it fell significantly after fermentation by various strains. This may be due to aldehyde molecules’ instability, which is reduced to alcohol or oxidized to acids when bacteria are present (37). Isobutyraldehyde, hydroxyacetaldehyde, and 3-furfural are primarily found in unfermented sweet potato slurry and are undetectable after fermentation. 5-hydroxymethylfurfural is the predominant aldehyde found in sweet potato slurry that has been fermented. Esters were not detected in unfermented sweet potato slurry, although their concentration increased to variable degrees after various microorganism fermentation. Esters are important components of flavor compounds, and increasing their content can impart a pleasant odor in fermented sweet potato slurry (38). The main esters after fermentation are glycidyl acrylate and diethyl phthalate. The level of alcohol in unfermented sweet potato slurry was rather low, and it was only greatly raised by *S. cerevisiae*-fermented sweet potato slurry, which reached 27.53%. This means that *S. cerevisiae* could convert sugars in sweet potato slurry to alcohols, mainly were ethanol and methanol (39). After fermentation, the content of ketones was significantly increased after the fermentation. Sweet potato slurry. The main ketones are 1,3-dihydroxyacetone and methyl-4 (H)-pyran-ketone. Except for *B.brevis*, the acid components in sweet potato slurry increased significantly after fermentation. After *B. coagulans* fermentation, the acid concentration was the highest among these strains, reaching 30.61%. This result was consistent with the trend of pH change after fermentation, showing that a high quantity of acid flavor substances was formed after the fermentation of *B. coagulans*, thereby significantly enhancing the flavor components of sweet potato slurry after fermentation (40).

## Conclusion

In this investigation, seven strains were applied to the fermentation of sweet potato slurry. The effects of several fermentation strains on the nutritional, functional components, and volatile taste compounds of sweet potato slurry was systematically investigated. The findings demonstrated that different fermentation strains could alter the pH, total sugar, total protein, total phenols, SDF, free amino acids, organic acid, and volatile taste compounds after fermentation compared to unfermented sweet potato starch processing slurry. The results demonstrated that the fermentation of sweet potato slurry by *A. niger* and *B. coagulans* could greatly increase protein, total phenolic content and volatile flavor, which is crucial for enhancing nutrient absorption and flavor considerably. These two strains have the potential to serve as ideal strains for enhancing the nutritional value of sweet potato fermentation slurry and enhancing its flavor and taste, hence enhancing their application value. Therefore, this study can provide ideas and theoretical support for developing a sweet potato slurry with several uses.

## Data availability statement

The raw data supporting the conclusions of this article will be made available by the authors, without undue reservation.

## Author contributions

LP: writing-original draft, data curation, investigation, methodology, formal analysis, resources, software, and visualization. C-JZ, ZB, W-ZT, and YZha: data curation, investigation, and methodology. Y-YL, YZho, and Y-YZ: formal analysis, resources, software, and visualization. A-ML, Y-CH, and G-HY: writing-review and editing, and supervision. MH: supervision. J-HH: writing-review and editing, resources, supervision, and project administration. All authors contributed to the article and approved the submitted version.

## Funding

This work was supported by High-Level Talents Research Fund of HAUT (Grant No. 2020BS064), Henan Province Science and Technology Research and Development Plan Joint Fund Project (222103810060), Henan Province Postdoctoral Research Project Funding (202103120), Henan Province Youth Science Fund Project (232300421266). This work was also financially supported by, Major Science and Technology Projects for Public Welfare of Henan Province in China (Grant No. 201300110300), Zhongyuan Scholars of Henan Province in China (192101510004), Zhongyuan Scholar Workstation Funded Project (ZYGZZ2021056, 224400510026), The Open competition Research Projects of Xuchang University (2022JBGS11), and Central Government Guides the Local Science and Technology Development Special Fund (Z20221341069).

## Conflict of interest

The authors declare that the research was conducted in the absence of any commercial or financial relationships that could be construed as a potential conflict of interest.

## Publisher’s note

All claims expressed in this article are solely those of the authors and do not necessarily represent those of their affiliated organizations, or those of the publisher, the editors and the reviewers. Any product that may be evaluated in this article, or claim that may be made by its manufacturer, is not guaranteed or endorsed by the publisher.
